# Cholecystokinin receptors in Atlantic salmon: molecular cloning, gene expression, and structural basis

**DOI:** 10.1002/phy2.69

**Published:** 2013-10-02

**Authors:** Raja M Rathore, Anna R Angotzi, Ann-Elise O Jordal, Ivar Rønnestad

**Affiliations:** Department of Biology, University of BergenN-5020, Bergen, Norway

**Keywords:** Appetite regulation, Atlantic salmon, cholecystokinin receptors, digestive physiology, gene expression, ligand binding, protein structure

## Abstract

The peptide hormone cholecystokinin (CCK) exerts a wide range of digestive and CNS-related physiological signaling via CCK receptors in brain and gut. There is very limited information available on these receptors in Atlantic salmon. The aim of this study was to characterize CCK receptors in gut and brain of salmon. We have identified and cloned one CCK-1 receptor and duplicates of CCK-2 receptor in salmon. The phylogenetic analysis indicates the existence of one common ancestor gene for all CCK receptors. CCK-1R mRNA is highly expressed in pancreas followed by midgut, hindgut, gallbladder, and stomach indicating an involvement in pancreatic regulation and gallbladder contractions. CCK-2R1/gastrin mRNA is expressed at high levels in midgut and at relatively low levels in stomach, gallbladder, and pancreas. We postulate CCK-2R1/gastrin receptor to have gastrin-related functions because of its distribution and abundance in gastro-intestinal (GI) tissues. CCK-2R2 is relatively abundant in brain but has low expression levels in gut tissues supporting the hypothesis for involvement in the gut-brain signaling. Major functional motifs and ligand interaction sites in salmon are conserved with that of mammals. This information will be instrumental for comparative studies and further targeting receptor activation and selectivity of biological responses of CCK in salmon.

## Introduction

Cholecystokinin (CCK) is a key regulatory peptide hormone that serves a wide variety of physiological actions. It is a prototype of a class of agents known as brain-gut peptides, functioning both as neuropeptide and gut hormone. In peripheral tissues, CCK is known to be involved in multiple biological processes such as pancreatic exocrine secretion, insulin release, and gut motility (Wank 1995; Tullio et al. 2000; Dufresne et al. 2006; Rehfeld et al. 2007; Staljanssens et al. 2011). CCK also affects cell growth (pancreas and various tumors). Within the brain, CCK actions elicit satiation, anxiety-related behavioral responses and stimulate growth proliferation signaling protein extracellular signal-regulated kinase 1/2:ERK1/2 (Yassin 1999; Piiper et al. 2003).

CCK exerts its biological actions by binding to specific G-protein-coupled receptors (GPCRs) known as CCK-1R and CCK-2R (formerly also termed as CCK-AR and CCK-BR, respectively). These receptors differ in their distribution and their affinities for natural agonists. CCK-1R is more predominant in the periphery especially in pancreas, gallbladder, and gastric mucosa whereas CCK-2R is more abundant in different parts of central nervous system (CNS) (Tullio et al. 2000). CCK-1R is highly selective for sulfated CCK analogues. Gastrin receptor in stomach is closely related to the CCK-2R subtype and they are often referred as combined CCKB/gastrin receptor as they show similar high affinity for both sulfated and nonsulfated peptide analogues (Lee et al. 1993; Dufresne et al. 2006).

For the CCK receptors in alimentary tract, comprehensive knowledge is available on their involvement in biological processes in mammals, while there is very limited information on CCK receptors in fishes (Furutani et al. 2013). The aims of this study were therefore to identify CCK receptors in Atlantic salmon and to elucidate their expression patterns in different gastro-intestinal (GI) compartments as well as in brain. In addition, we have assessed the putative phylogenetic relationships existing among CCK-R genes and predicted the 3D structure, interacting motifs and the agonist-binding sites of these receptors. Altogether these studies provide new insights into the conservation of CCK-R genes structure and most likely functions during evolution, as well as a better understanding for further studies in receptor activation and downstream biological responses of CCK.

## Material and Methods

### Specimen and tissues

Atlantic salmon (average body weight 165 ± 3.97 g) were reared at the Bergen High Technology Center (Norway), in indoor tanks (500 L rearing volume) supplied with continuous water flow (2.5 L min^−1^, 8°C) and fed ad libitum on a commercial diet (EWOS, Norway) for 3 weeks, as described by Rønnestad et al. (2010). All handling of animals followed the Norwegian Animal Research Authority (NARA) guidelines for experimental purposes. At the end of the experiment (9 am, after feeding), fish were randomly sampled and sacrificed by an overdose of MS-222 (3-aminobenzoic acid ethyl ester; Sigma, St. Louis, MO). Tissue samples were collected in liquid nitrogen and kept at -80°C until further analysis.

### Gene expression studies

#### RNA extraction

Total RNA was extracted using TRI reagent (Sigma, St. Louis, MO). Isolated RNA was further purified by DNAse treatment (Turbo DNA free kit, Ambion, Life Technologies Corporation, CA). First strand cDNA was synthesized from 4 μg of total RNA using oligo(dT)20 primer and Superscript III (Invitrogen, Carlsbad, CA) according to manufacturer's protocol.

#### Identification and cloning of *Salmo salar* CCK-1R, CCK-2R1, and CCK-2R2 genes

Zebrafish CCK-1R and CCK-2R open reading frames (ORFs) (acc.no. NM_001085808.1 and XM-002663315.2) were used as a query against the codgenome project (http://www.codgenome.no/) *Salmo salar* data by using translated tblastn search (http://www.codgenome.no/blast/blast_new.php) and the Trace Archive Nucleotide (http://blast.ncbi.nlm.nih.gov/Blast.cgi). CCK-1R sequence fragments were identified in scaffolds gnl|ti|2263327594 and gnl|ti|2287331943 while CCK-2R1/gastrin and CCK-2R2 in scaffolds gnl|ti|2279893979, gnl|ti|2263101519, and gnl|ti|2319219166gnl|ti|2317620741, respectively. ORF and 5' untranslated regions were obtained by real-time polymerase chain reaction (RT-PCR) and rapid amplification of cDNA ends (RACE) method according to the manufacturer's instructions (Marathon™ cDNA Amplification Kit; Clontech, Mountain View, CA). Finally, cDNA fragments containing the complete coding regions of CCK-1R and CCK-2Rs were amplified using LongRange PCR Kit (Qiagen, Hilden, Germany).

To amplify fragments within the range of 1.5 kb, PCR reactions were performed using GoTaq PCR reagents (Promega, Madison, WI), according to manufacturer's instructions. PCR conditions were as follows: initial activation of the Taq-DNA-Polymerase for 5 min at 94°C, followed by 33 cycles of denaturation for 25 sec at 94°C, annealing for 30 sec at 58°C, and extension for 90 sec at 72°C. The program ended with an incubation of 7 min at 72°C. Gel-purified products were cloned into a pCR4-TOPO vector using TOPO TA Cloning® Kit (Invitrogen). Sequencing was performed at University of Bergen Sequencing facility using BigDye Terminator v3.1 chemistry in ABI PRISM377 DNA sequencer (Applied Biosystems, Foster City, CA).

Sequences were analyzed by homology across species with the basic local alignment search tool (blast; http://www.ncbi.nlm.nih.gov) to confirm gene orthology. Subsequent sequence analysis using other bioinformatics tools such as clustalW alignment revealed the finding of two paralogue duplicates (CCK-2R1/gastrin and CCK-2R2) of the CCK-2R gene and one single copy of the CCK-1R gene. The lists of primers used to identify these genes are given in Table 1.

**Table 1:** Oligonucleotide primers used in this study

Primers	Sequences	Purpose
cckar7 Fw	GTACCTGGCAAACCAAGTCCCAT	cckar first PCR
cckar 9 Rv	GATGGAGAAAGGAAGCTAAGCCTG	cckar first PCR
cckbr 5 Fw	TCCACCTTCAGCCTGGTTGCCATA	cckbr first PCR
cckbr 9 Rv	AGTCAGTCAATGGTTCCTCCCTCA	cckbr first PCR
cckar 10 Fw	AGTGATGTCATCCAGCAGTCCT	cckar RT-PCR
cckar 11 Rv	TCAGCTTGGACTTGTTGTTCTCTCT	cckar RT-PCR
cckbr 10 Fw	TCCACCTTCAGCCTGGTTGCCATA	cckbr RT-PCR
cckbr 11 Rv	ACGTAGCATCCATCACCGTCGT	cckbr RT-PCR
cckar 10 Fw	AGTGATGTCATCCAGCAGTCCT	cckar Q-PCR
cckar 15 Rv	CTGAAGGTAGCAGCCGTCGTTGT	cckar Q-PCR
cckbr _1_ 16 Fw	ATCTCCACCTTCAGCCTGGTTGCCATA	cckbr-1 Q-PCR
cckbr _1_ 18 Rv	ACACATGCGTGCGATGGTGATGTTGT	cckbr-1 Q-PCR
cckbr _2_ 21 Fw	TCATCGACGGCAACCACGTCCAAATT	cckbr-2 Q-PCR
cckbr _2_ 23 Rv	AGGAAACCAGCACAGGAAGAACAGAGCA	cckbr-2 Q-PCR
Elf1α Fw	GAGAACCATTGAGAAGTTCGAGAAG	Q- PCR reference
Elf1α Fw	GCACCCAGGCATACTTGAAAG	Q- PCR reference
5′RACE ccka P_1_	CATGGGACTTGGTTTGCCAGGTACG	cckar 5′RACE
5′RACE ccka P_2_	GATTGAAGGTAGAGACACTCACCGAGAT	cckar 5′RACE
5′RACE cckb P_1_	TATGGCAACCAGGCTGAAGGTGGA	cckbr 5′RACE
5′RACE cckb P_2_	AGAGCCCTATGGGAACGCATGGAA	cckbr 5′RACE
cckbr Lr fw	AGATCCACTCAACTCATGAACCCATACTG	Long range PCR
cckbr Lr Rv	AGTCAGTCAATGGTTCCTCCCTCA	Long range PCR
cckar-2 Fw	AAGAGAGAACAGCAAAAATGGAACCA	Long range PCR
cckar-2 Rv	GATGGAGAAAGGAAGCTAAGCCTG	Long Range PCR
Cckbr2d fw	GCACATAGCACGAATATAATGTGC	Long range PCR
Cckbr2f rv	CCAGTCAGTCAATGGTTCCTC	Long range PCR

#### Real-time quantitative PCR

mRNA expression levels of CCK-1R, CCK-2R1 and CCK-2R2 and of an endogenous housekeeping gene encoding for Elongation factor 1-alpha (Elf1α; Genbank accession No. AF321836) were quantified using quantitative PCR (qPCR) analysis on the CFX-96 RT-PCR detection system platform (Bio-Rad, Hercules, CA), by Power SYBR Green PCR kit (Applied Biosciences, UK), in a final volume of 25 μL per reaction. qPCR analysis was conducted on cDNA that was reverse transcribed using RNAs pooled from six fish. To minimize sample variability, all samples were run in triplicates. The qPCR conditions were as follows: 94°C for 5 min and 42 cycles at 94°C for 30 sec, 60°C for 30 sec, and 72°C for 30 sec. Absence of primer dimers and nonspecific products was verified in every qPCR assay by melting curve analysis (temperature reading every 0.2°C from 60°C until 95°C).

The choice of Elf1α as a reference gene was based on a previous study, where amplification of this gene showed a steady-state level of expression among different segments of salmon brain and intestine (Murashita et al. 2009). Expression data of Elf1α were used for normalization. Standard curves (twofold dilution series of cDNA) were generated for target and Elf1α genes by plotting the cycle threshold (Ct) obtained in qPCR analysis versus the logarithm of input quantity of RNA and performing a linear regression (Bustin 2000). The threshold was consistently set for 0.10 and analyzed using CFX manager software. The data were exported in to Microsoft Excel for further analysis. Ct values of triplicates were processed using *Q-gene* software (Simon 2003).

#### Phylogeny

Phylogenetic analysis was performed by PhyML using software Phylogeny.fr (Dereeper et al. 2008) run with a maximum likelihood method (aLRT) statistical test (Jagerschmidt et al. 1998) of branch support to build a tree with 100 bootstrap replicates, based on available CCK-1R and CCK-2R-like sequences retrieved from Genbank (NCBI). Complete amino acid sequences for each protein were used to construct the phylogenetic tree. CCK-R-like sequences from invertebrates [Honey bee (*Apis mellifera*), Beetle (*Tribolium castaneum*), and Nematode (*C. elegans*)] were included as outgroups.

#### In silico analysis of protein sequence, structure modeling, and ligand docking

Putative transmembrane domains were predicted using TMHMM 2.0 server (Denmark), which is part of the Simple Modular Architecture Research Tool. Transmembrane protein 2D topology was designed using RbDe tool of Institute of computational biomedicine, Cornell University (Skrabanek et al. 2003).

Three-dimensional models for CCK-1R and CCK-2R1/gastrin were created and viewed using Rasmol. Because the alignment between CCK-2R1/gastrin and CCK-2R2 gave 90% similarity including the position of functional motifs, we have only modeled CCK-2R1/gastrin. PDB-ID 2KS9 chain A determined using nuclear magnetic resonance was found to be a good template (Identity ∼25% & Similarity ∼40%) for modeling using BLAST against protein data bank (PDB). This template satisfied the additional constraint of possible disulfide bond between the cystine residues in the studied protein sequence. The modeled structure with I-TASSER for CCK-1R and CCK-2R1/gastrin has TM-score ∼0.56 ± 0.15 and ∼0.58 ± 0.14 with RMSD score ∼9.9 ± 4.6 and ∼9.6 ± 4.6 Å, respectively. SWISS-MODEL reported Anolea and Gromos scores in favorable negative range for most of the modeled residues with final energy lower than 3500 kJ/mol in average. Furthermore, the Modeler showed mean DOPE score of ∼7200 and GA341 ∼0.55 on average. WHAT-IF analysis showed the possibility of cystine bridges between residue positions 119, 208 and 130, 213 for CCK-1R and CCK-2R1/gastrin, respectively. Docking with natural ligand cholecystokinin-8 (PDB ID 1 D 6G chains B-CCK-8) was performed using HADDOCK and based on the HADDOCK scores, potential ligand interacting residue segments were considered. For both CCK-1R and CCK-2R1/gastrin the residues of extracellular loop 2 (ECL2) and ECL3 were found as the best candidates. Potential ligand interaction residues were predicted using LigPlot+. The sequences were aligned to find out the conserved potential residues and domains in Atlantic salmon. The software programs used for all the analyses in this study are listed in Table 2.

**Table 2:** Bioinformatics tools and databases used for sequence and structural analysis

Purpose	Programmes used
Primary sequence analysis	BLAST (http://www.ncbi.nih.nlm.gov)
	EMBOSS (http://emboss.sourceforge.net/)
	CLUSTALW (http://www.ebi.ac.uk/services)
	I-TASSER (http://zhanglab.ccmb.med.umich.edu/I-TASSER/)
Putative transmembrane prediction	(http://www.cbs.dtu.dk/services/TMHMM/)
Residual model	RbDe (http://icb.med.cornell.edu/services/rbde/diagrams)
3D Modeling	Modeller (http://www.salilab.org/modeller/)
	SWISS-MODEL (http://swissmodel.expasy.org)
Protein 3D visualization	Rasmol (http://openrasmol.org/)
RMSD calculation	DALI (http://ekhidna.biocenter.helsinki.fi/dali_lite/start)
Cysteine bridge analysis	WHAT-IF (http://swift.cmbi.ru.nl/servers/html/listcys.html)
Protein motif analysis	Regex engine inbuilt in PERL (http://www.perl.org/).
Ligand docking	HADDOCK (http://haddock.science.uu.nl/services/HADDOCK/haddock.php)
Interacting residues for ligand binding	LigPlot+ (http://www.ebi.ac.uk/thornton-srv/software/LigPlus/)
Phylogenetic tree building	PhyML http://www.phylogeny.fr/

## Results

### Cloning and sequence analysis

This study reports the identification of both subtypes of cholecystokinin receptors (CCK-1R and CCK-2R). Furthermore, we found duplicates of CCK-2R gene, named CCK-2R1/gastrin and CCK-2R2. The accession numbers assigned by NCBI for CCK-1R is JX017294 and that for CCK-2R1/gastrin and CCK-2R2 are JX017295 and JX017296, respectively.

Using bioinformatics and RACE PCR approaches, we obtained full-length products for all genes studied here. The isolated product for CCK-1R cloned from pancreas cDNA, has an ORF of 1418 bases including a CDS encoding a translated protein of 461 amino acids with a molecular mass of 51.29 kDa. The ORF of CCK-2R1/gastrin cloned from midgut cDNA consisted of 1608 bases and comprised a CDS of 1359 bases encoding for a putative protein of 453 amino acids. CCK-2R2 transcripts cloned from brain tissues contained an ORF of 1426 bases and encoded for a protein of 449 amino acids. Molecular mass of both CCK-2R receptors was predicted to be of ∼50 kDa. Sequence homology blast search confirmed gene orthology with CCK receptor genes of other species.

Alignment of salmon CCK-1R showed 73% amino acid sequence similarity with zebrafish and Nile tilapia CCK-1R genes and 63% similarity with the human ortholog (Table 3). CCK-2R1/gastrin and CCK-2R2 showed 90% amino acid sequence similarity among themselves and shared 70% similarity with zebrafish and Nile tilapia CCK-2R gene while about 50% with human and mouse orthologs (Table 4).

**Table 3:** Amino acid sequence identity (%) between newly identified salmon CCK-1R and known CCK-1R orthologs

	Salmon CCK-1R
Zebrafish CCK- 1R	73
Nile tilapia CCK-1R	73
Pufferfish CCK-1R like	80
Frog CCK-1R	59
Chicken CCK-1R	63
Human CCK-1R	63
Mouse CCK-1R	55

**Table 4:** Amino acid sequence identity (%) between newly identified salmon CCK-2R and known CCK-2R orthologs

	Salmon CCK- 2R1/gastrin	Salmon CCK-2R2
Salmon CCK-2R1/gastrin	–	90
Salmon CCK-2R2	90	–
Zebrafish CCK-2R	70	68
Nile tilapia CCK-2R	71	71
Pufferfish- CCK-2R like	70	70
Frog CCK-2R	55	55
Chicken CCK- 2R	55	56
Human CCK- 2R	49	50
Mouse CCK-2R	47	47

### Phylogeny

**Figure 1:**
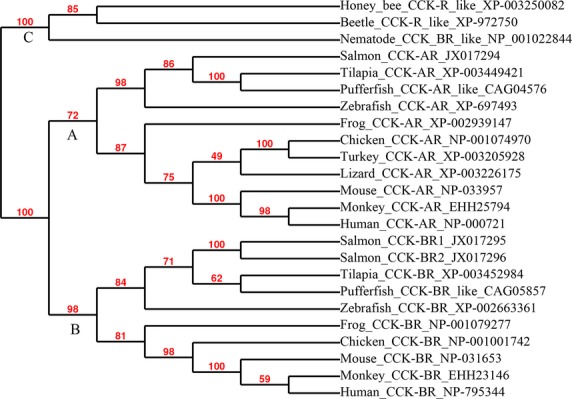
Phylogenetic analysis of CCK-1R (CCK-AR) and CCK-2R (CCK-BR). Numbers at nodes indicates the bootstrap value, as percentages, obtained for 100 repetitions. Gene accession number for each species is given along with the species name. Branch A, B and C represents vertebrate CCK-1R, vertebrate CCK-2R, and invertebrate CCK-R-like genes, respectively.

The phylogenetic tree indicated that invertebrate CCK-R genes clearly form a separate clade with 100% boot strap value (clade C in Fig. 1). Both receptor types cluster together with the respective CCK subtype receptor orthologs in two main clades with good boot strap support (clades A and B in Fig. 1; 72 and 98%, respectively). Interestingly, CCK-2R1/gastrin and CCK-2R2 genes are more closely related to each other (boot strap support 100%) than either of them is to their orthologs as predicted if they arose from recent lineage-specific genome duplication. These results are in good agreement with the concept of traditional taxonomy.

### Structural analysis

**Figure 2:**
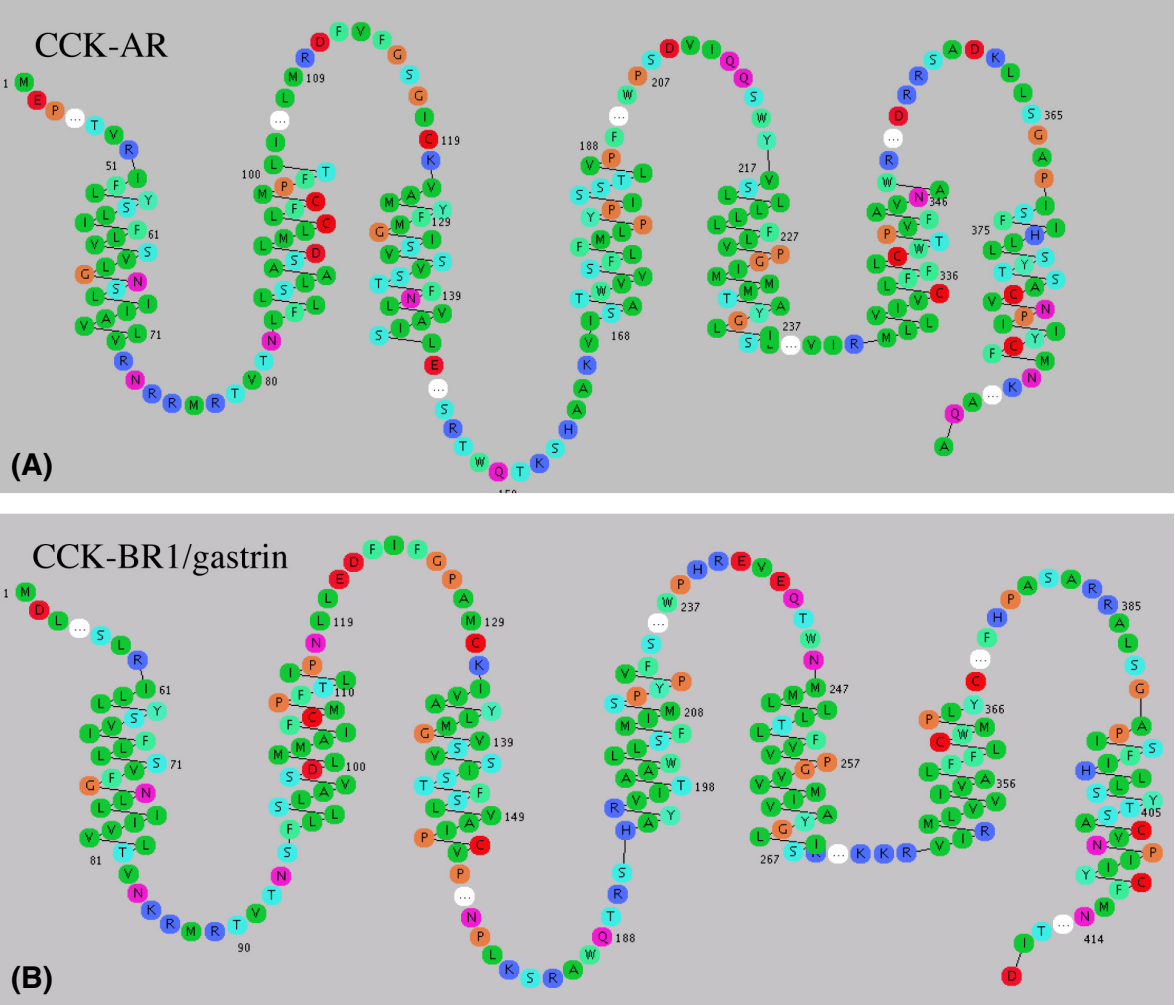
Residual model diagram of salmon CCK-1R (A) and CCK-2R1 (B).

**Figure 3:**
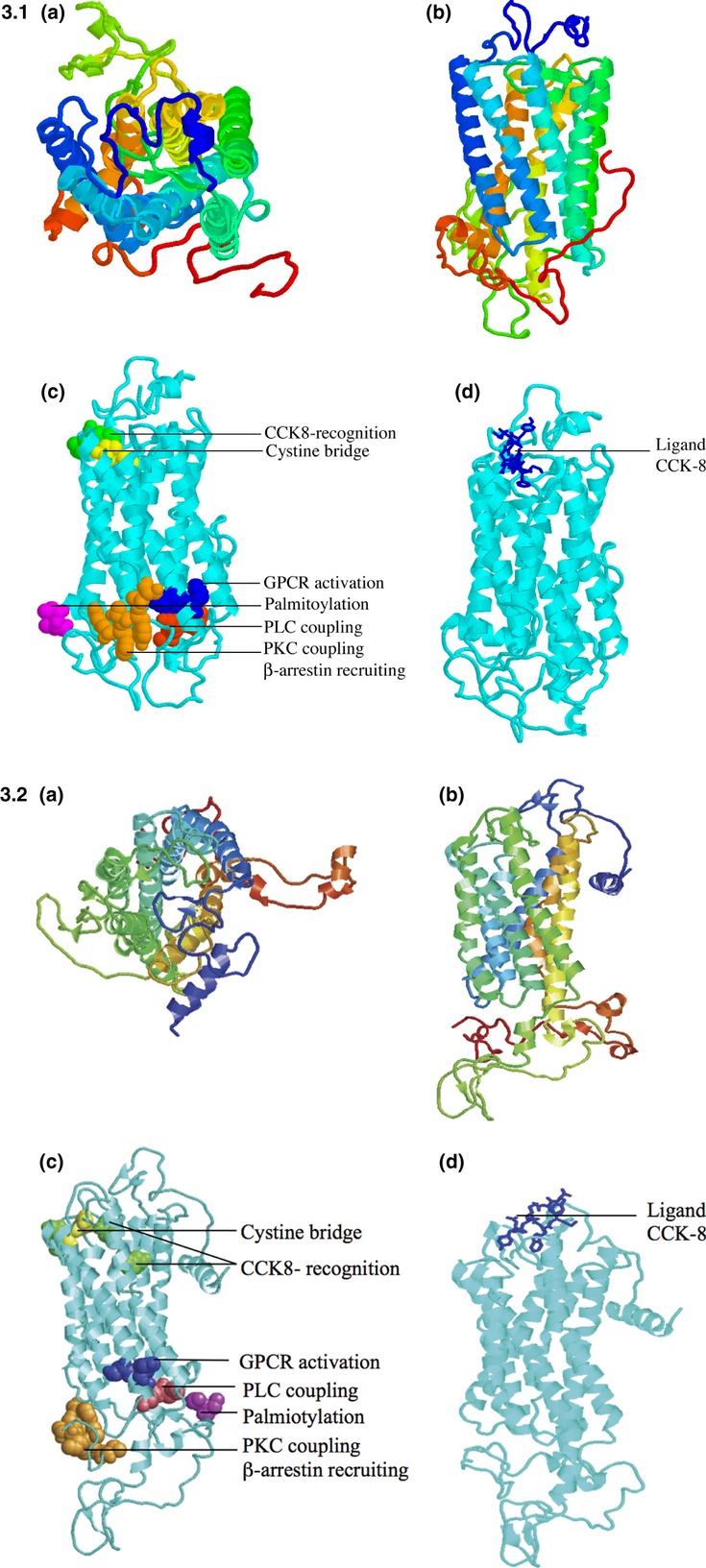
Rasmol visualization of 3D model of Atlantic salmon CCK-1R (3.1) and CCK-2R1/gastrin (3.2) Each figure is shown in four panels: (a). Top view (b). Front view (c). Front view with predicted motif positions (d). CCK-8 and CCK-R complex.

**Figure 4:**
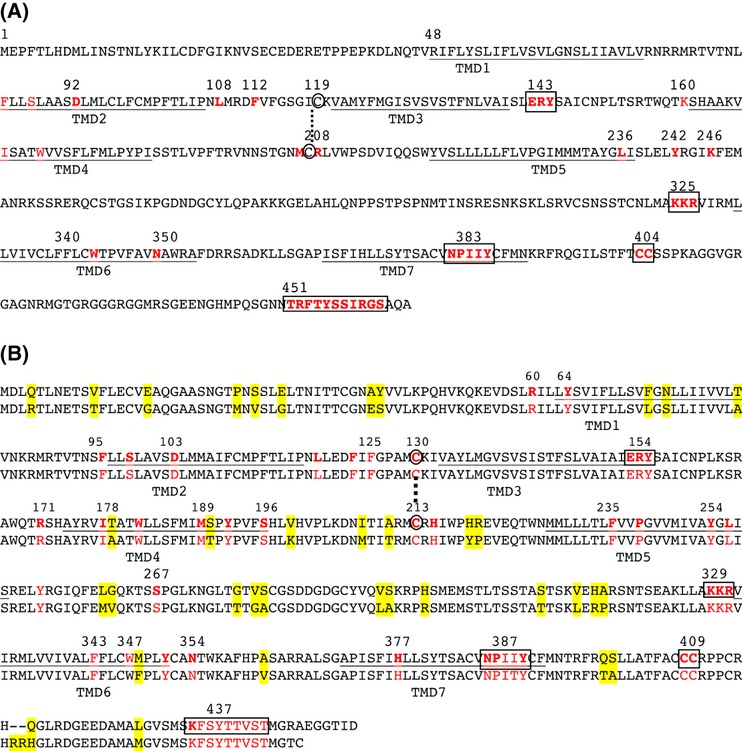
Predicted functional motifs and affinity determinants in the amino acid sequence of Atlantic salmon CCK-1R (A), and CCK-2R1/gastrin and CCK2-R2 (B) CCK-2R1 sequence is presented on the top and CCK-2R2 below. Functional motifs are marked red with each residual position given above the sequence. Trans-membrane domains are underlined and Cystine bridge position is circled (for description see Table 5).

**Figure 5:**
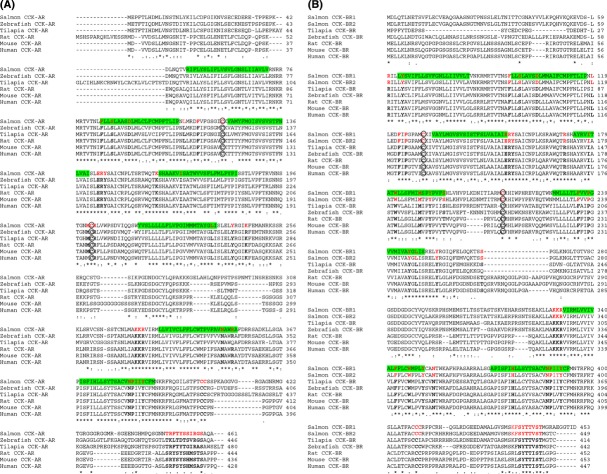
Alignment of CCK-1R (CCK-AR) amino acid sequences (A), CCK-2R1/gastrin and CCK-2R2 (CCK-BR) (B) with known functional domains of other vertebrates. Trans-membrane domains are marked in green. Residues marked bold represent functional motifs and those in circles cystine bridge residues.

**Figure 6:**
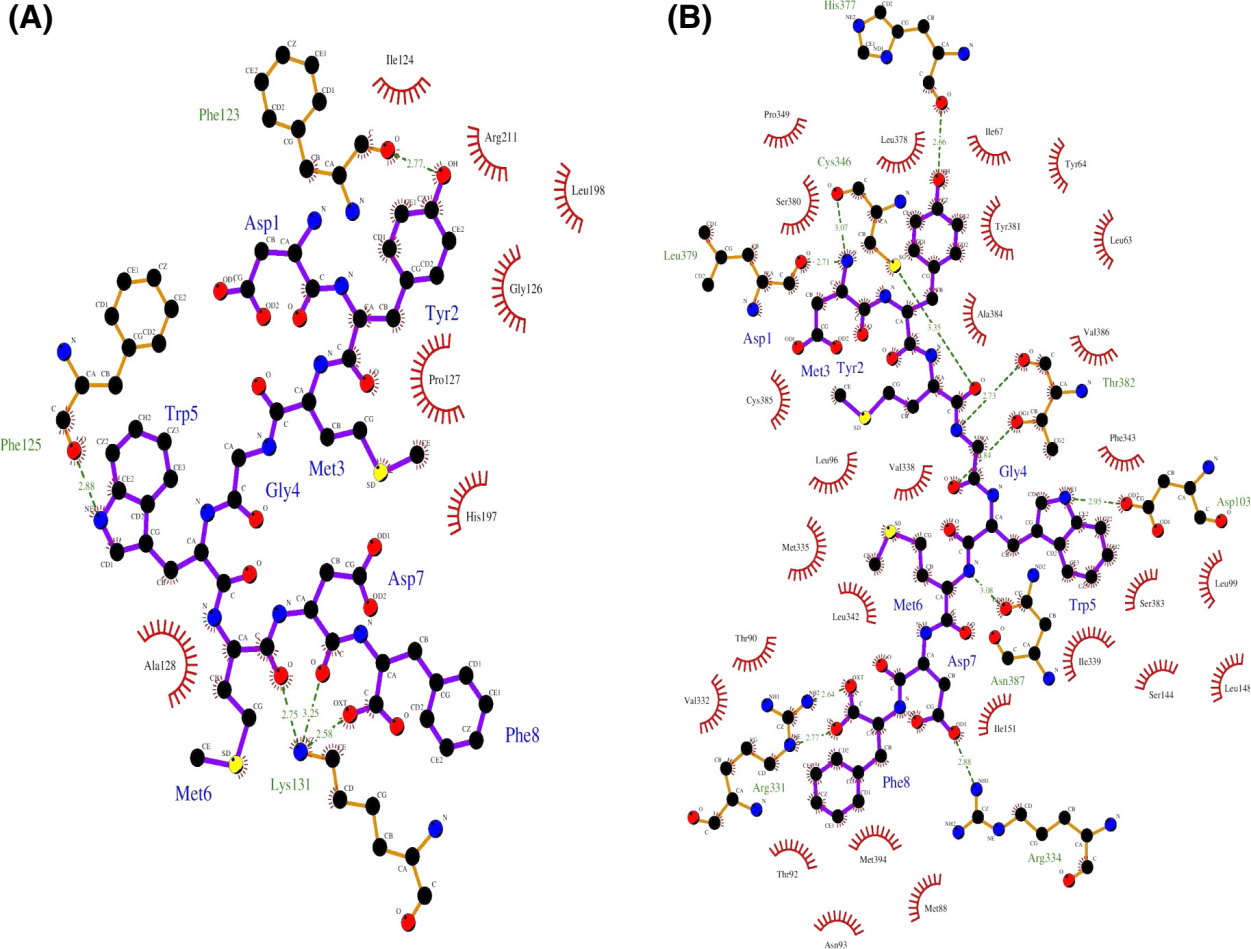
Ligplot+ predictions of first Extracellular loop (A) and second Extracellular loop (B) of Atlantic salmon CCK-2R1/gastrin. Figure shows complex of unsulfated CCK8 (molecular model in dark blue) with interacting residues (marked in green).

Residual modeling of salmon CCK receptors clearly suggests that these proteins belong to seven-transmembrane GPCR family (Fig. 2). The 3D models generated taking PDB ID 2KS9 as template confirmed this assignment (Fig. 3). WHAT-IF analysis showed the possibility of disulfide bond among cystine residues between first and second extracellular loop (Fig. 4), further confirmed with prediction from ScanProsite database. The amino acid sequences were aligned with zebrafish, Nile tilapia, rat, mouse, and human to identify potentially conserved functional motifs. Functional motifs such as E/DRY, NPxxY, PLC, PKC, CRAC, CCM, and Palmitoylation domains (Kristiansen 2004; Potter et al. 2012) were found conserved in residual positions to the human CCK receptors (Fig. 5A and B). A detailed account of motif location corresponding to human and its function is given in Table 5. Sequence alignment confirmed the position of human CCK-8 affinity determinant residues Leu-116, Phe-120 of first ECL; Tyr-61of TMD1 (trans-membrane domain 1); Met 186, Tyr 189, Thr 193 of TMD3; His 207 of second extracellular loop were conserved for both the salmon receptors studied here (Fig. 5). Ligplot+ docking analysis data further supported possible interaction of unsulfated CCK-8 with Phe-125 in first extracellular loop and His-377 in TMD7 for CCK-2R1/gastrin (Fig. 6).

**Table 5:** Functional motif and residue positions of Atlantic salmon CCK receptors in relation to human

Motif	CCK-1R		CCK-2R		Function	Reference
	
	Posistion human	Posistion salmon	Posistion human	Posistion salmon		
Cystine bridge	C114-C296 (ECL1-ECL2)	C119-C208 (ECL1-ECL2)	C127-C205 (ECL1-ECL2)	C130-C213 (ECL1-ECL2)	Disulfide bridge	(Dufresne et al. 2006)
E/DRY	138-139 (ICL2)	143-145 (ICL2)	151-153 (ICL2)	154-156 (ICL2)	G protein activation, receptor confirmation	(Fourmy et al. 2002)
NPxxY	366-370 (TMD7)	383-387 (TMD7)	386-390 (TMD7)	387-391 (TMD7)	Receptor internalization	(Miller and Gao 2008)
CC	387-388 (N-terminus)	404-405 (N-terminus)	408-409 (N-terminus)	409-410 (N-terminus)	Palmitoylation site	
D	87 (TMD-2)	92 (TMD-2)	100 (TMD-2)	103 (TMD-2)	PLC coupling site	(Dufresne et al. 2006)
FKKR	F 218 (TMD5)308-310 (ICL-3)	F213 (TMD5)325-327 (ICL-3)	F227 (TMD5)328-330 (ICL-3)	F235 (TMD5)273-275 (ICL-3)		
KKR	308-310 (ICL-3)	325-327 (ICL-3)	328-330 (ICL-3)	273-275 (ICL-3)		
R/K-x-S/T	415-417 (N-terminus)	449-451 (N-terminus)	435-437 (N-terminus)	435-437 (N-terminus)	PKC phosphorylation	(Magnan et al. 2011)
Serine-threonine motif	418-423 (N-terminus)	448-458 (N-terminus)	437-443 (N-terminus)	437-443 (N-terminus)	β- arrestin recruitment	
CRAC	L231/Y237/K241	L236/Y242/K246	L240/Y246/R250	L248/Y254/Q 258	Cholesterol recognition/interaction	(Potter et al. 2012)
CCM	K155/I162/W166 F79/S82	K160/I167/W171 F84/S87	R168/I175/W179 F93/S96	R171/I178/W182 F95/S98	Cholesterol consensus motif	
Agonist interacting	L50/I52/L53 (TMD1)	L55/I56/L58 (TMD1)	R57/Y61 (C-terminus)	R60/Y64 (C-terminus)	CCK peptide recognition and transduction	(Silvente-Poirot et al. 1998)
	C94 (TMD2)	C99 (TMD2)	L116/F120 (ECL1)	L119/F123 (ECL1)		
	F107 (ECL1)	F 112 (ECL1)					
	M 121/V125 (TMD3)	M126/R130 (TMD3)	H 207 (ECL2)	H 215 (ECL2)		(Foucaud et al. 2008)
	M195/R197 (ECL2)	M200/R202 (ECL2)	M186/Y189/T193 (TMD4)	M189/Y192/S196 (TMD4)		(Gigoux et al. 1999; Gale et al. 2003)
	F 218 (TMD5)	F (TMD5)	F227/P230 (TMD5)	F235/P238 (TMD5)		(Jagerschmidt et al. 1998)
	W 326/F330 N333/R336 (TMD6)	W343/F347 N350/R353 (TMD6)	F342/W346 (TMD6)	F343/W347 (TMD6)		
	S348 (ECL3)	S 365 (ECL3)	N353 (ECL3)	N354 (ECL3)		(Gigoux et al. 1999; Ren et al. 2003)
	I352/Y 356 (TMD7)	I369/Y377 (TMD7)	H376 (TMD7)	H377 (TMD7)		(Miller and Gao 2008)

### Gene expression and tissue distribution

**Figure 7:**
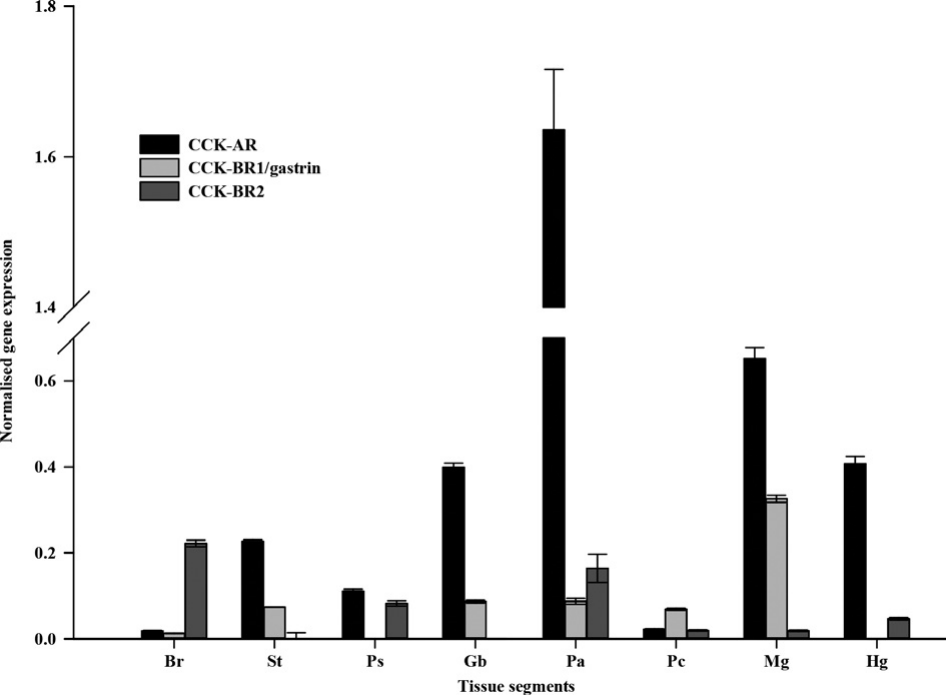
Tissue distribution and relative expression levels of the CCK receptors in Atlantic salmon. CCK receptor mRNA levels were normalized with Elf1a gene. Error bars represent standard error of the mean (*n* = 3). Br, brain; St, stomach; Ps, pyloric sphincter; Gb, gall bladder; Pa, pancreas; Pc, pyloric ceca; Mg, midgut; Hg, hindgut.

The mRNA tissue distribution of the three CCK receptors in eight tissues from Atlantic salmon was assessed using qPCR approach (Fig. 7). CCK-1R mRNA expression was significantly abundant in pancreas followed by midgut, hindgut, and gallbladder. Relatively low levels of CCK-1R transcripts were found in pyloric sphincter and pyloric ceca while the lowest levels were found in brain. A significantly higher level of CCK-2R1/gastrin was seen in midgut compared to the other organs. The levels of CCK-2R1/gastrin in stomach, gallbladder, and pancreas were not significantly different from each other. CCK-2R1/gastrin mRNAs were not detected in pyloric sphincter and hindgut. CCK-2R2 mRNA expression was most abundant in brain followed by exocrine pancreas, pyloric sphincter, and hindgut. CCK-2R2 mRNA transcripts were not found in stomach and gallbladder.

**Figure 8:**
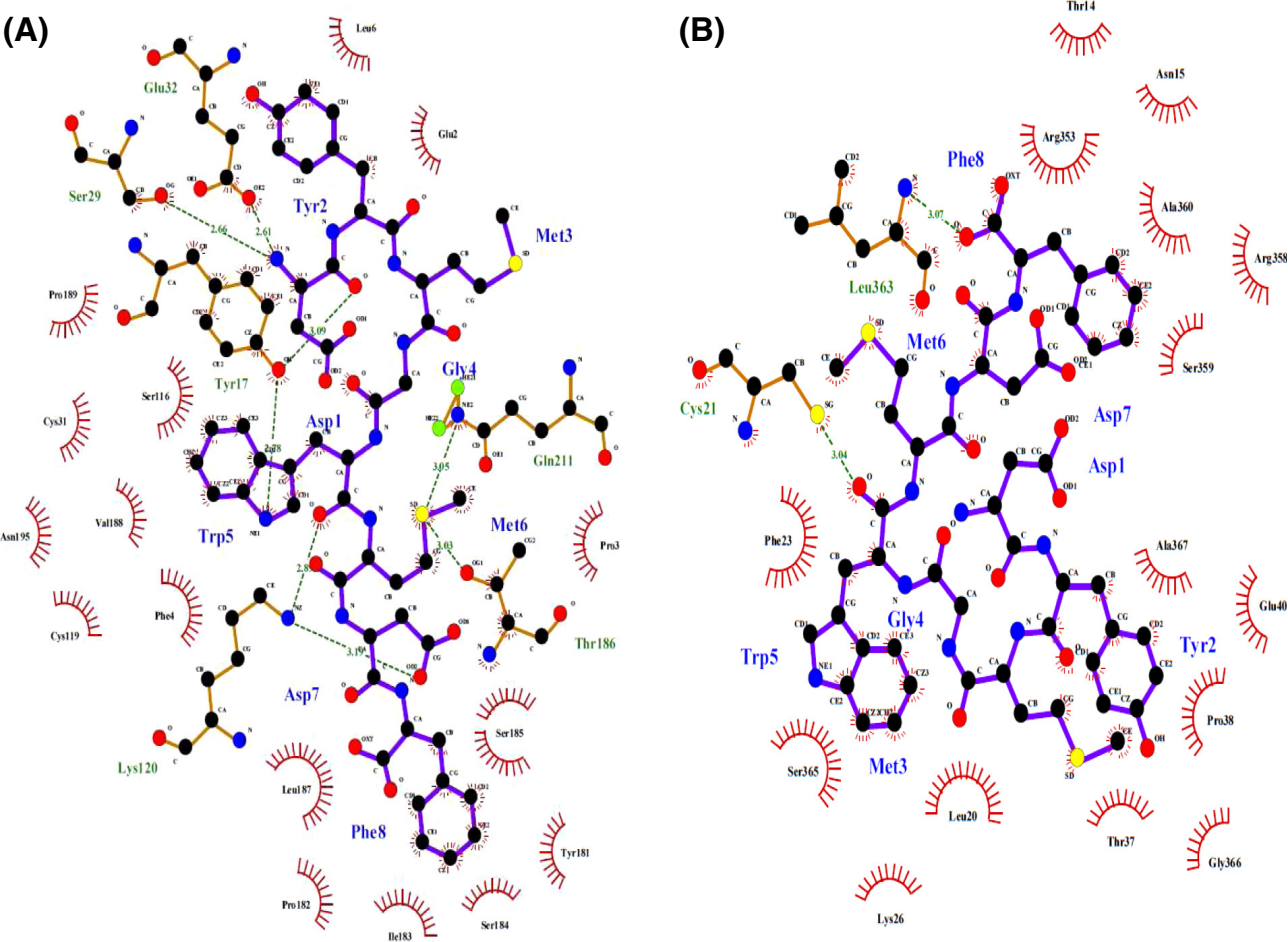
Ligplot+ prediction of 1^st^ Extracellular loop (A) and 2^nd^ Extracellular loop (B) of Atlantic salmon CCK-1R. Figure shows complex of unsulfated CCK8 (molecular model in dark blue) with interacting residues (marked in green).

## Discussion

### Sequence and phylogeny

The predicted evolutionary relationship of CCK-R and CCK-R-like receptors was evaluated using phylogenetic analysis with annotated ortholog sequences from the Genbank database. In our phylogenetic tree, vertebrate and non vertebrate CCK-R fall into separated clusters and a clear evolutionary split between the CCK-1R and CCK-2R is observed in the vertebrate group (Fig. 1). This is consistent with Salmon CCK-2R1/gastrin and CCK-2R2 showing 47 and 49% amino acid sequence homology with CCK-1R, respectively. Thus, although CCK-1R and CCK-2R genes are predominantly conserved among species, they show important functional divergences and exhibit different affinities for gastrin and CCK ligands (Yassin 1999). Besides, positions of both receptors in the tree pinpoint Nile tilapia as the closest relative to salmon. However, because only a limited number of teleost CCK-R sequences are currently available, this aspect remains to be explored further.

Duplicated genes are frequently found in teleosts as result of the fish-specific whole-genome duplication (FSGD, 3R duplication) that occurred about 350 million years ago (Meyer and Schartl 1999; Jaillon et al. 2004). An additional genome duplication event was postulated to occur in the last common ancestor of salmonids (4R duplication), resulting in a pseudotetraploid karyotype with multiple gene copies (Ohno 1970; Moghadam et al. 2005; Kurokawa and Murashita 2009). CCK-2R1/gastrin and CCK-2R2 paralogues share rather high amino acid conservation (90% similarity) suggesting that they most probably are recently duplicated genes following the 4R duplication of salmonids (Table 4). Furthermore, we postulate that salmon CCK-2R1/gastrin is the ortholog of mammalian gastrin receptor due to its prominent expression in alimentary-tract tissues and also due to the fact that CCK-2R2 is clearly predominant in brain.

### Structure and motif analysis

CCK receptors belong to the β-group of rhodopsin receptors or to the subfamily A6 in the A-F classification of GPCRs (Staljanssens et al. 2011). The residual and 3D structural modeling of salmon CCK-1R and CCK-2R1/gastrin was predicted to be typical of the GPCR superfamily by having seven TMDs (Fig. 3). We have verified the conventional characteristic signature sequences in human that provide the appropriate architectural motifs, to achieve the preferred structure for proper activity and regulation of the receptor. The alignment of CCK-1R and CCK-2R1/gastrin with respective ortholog sequences (Fig. 5A and B) clearly suggest that all these functional motifs, including their respective positions, are conserved in salmon for both the receptors. This is also the case for the signal transduction motifs PLC and PKC (refer to Table 5 for details).

### Ligand affinity

We were also interested to analyze ligand interacting regions in the sequences of salmon CCK-R genes. We have considered important residues and their positions for ligand affinity based on human and cell based studies. It is well known that CCK receptors provide a common structural basis for affinity to their natural ligand CCK, known to be present in several molecular variants. In humans the octapeptide CCK-8 (Asp-Tyr(SO_3_H)-Met-Gly-Trp-Met-Asp-Phe-NH_2_) has the highest activity (Escrieut et al. 2002). CCK-1R has a greater affinity for sulfated CCK than for sulfated or unsulfated gastrin and requires at least the C-terminal heptapeptide as requisite for binding and to elicit biological activities. On the other hand, CCK-2R/gastrin also binds to gastrin since this has the same C-terminal tetrapeptide and does not distinguish between gastrin and CCK, whether sulfated or not (Shulkes and Baldwin 1997; Dufresne et al. 2006; Staljanssens et al. 2011). Cell mutational studies suggest that all the amino acids determined to affect CCK-8 affinity in the CCK-2R protein are not necessarily important for CCK-8 binding in the CCK1R. However, two residue positions (Leu and Phe) of ECL1 seem to affect CCK-8 affinity in both CCK-2R and CCK-1R (Silvente-Poirot et al. 1998). These residues are conserved in salmon for both CCK-1R and-2R proteins (Fig. 5A and B).

Human CCK-1R residues in ECL 2 (Met and Arg) and TMD6 (Arg and Asn) that account for the selectivity and receptor-ligand stability for sulfated versus nonsulfated ligand, are found conserved with salmon. Interestingly, other reported signature residues for sulfated analogue binding (Trp39, Gln40) in human CCK-1R that interact with N-terminal moiety of CCK (Kennedy et al. 1997) are not found in salmon and suggest possible differential N-terminal binding mechanisms for sulfated CCK in this species. This is also reflected in the Ligplot+ analysis for CCK-1R with unsulfated CCK-8, where the prediction plot did not show any significant ligand-receptor interaction as per agreement with the available literature (Fig. 8A and B). This suggests that salmon CCK-1R may also have a preference for sulfated analogs over unsulfated ones as that in mammals. However, these aspects are subjected to further conformational studies.

For CCK-2R, experimentally determined putative CCK-8 affinity determinants in TMD 1 (Tyr), TMD4 (Met, Tyr, Thr), TMD 6 (Asn), and residues in ECL2 and ECL3 are all conserved with the position of human CCK-2R (Silvente-Poirot et al. 1998; Anders et al. 1999; Gigoux et al. 1999; Bläker et al. 2000; Gouldson et al. 2000; Giragossian and Mierke 2001; Gale et al. 2003). The Ligplot+ analysis of salmon CCK-2R1/gastrin predicts interaction of CCK-Tyr with salmon His-377 in TMD7 (Fig. 6). This is in agreement with earlier reports based on site-directed mutagenesis data for 3D model of the human CCK-2R and CCK complex (Foucaud et al. 2008). Another study on human models reported that CCK-Trp binds to Phe-120 located in the hydrophobic/aromatic pocket formed by residues from TMD3,7 and from the first extracellular loop (Foucaud et al. 2008). The alignment of amino acid sequence suggests the conservation of residual position between human Phe-120 and salmon Phe-123. Our ligand docking analysis from Ligplot+ predicts that Phe-123 in the first extracellular loop of salmon CCK-2R1/gastrin interacts with CCK-Tyr while residue Phe-125 with CCK-Trp (Fig. 6). However, salmon Phe-125 is also found to be conserved in human Phe-122 (Fig. 5B). As these data are based on predictions, further biological studies are essential to establish the exact interacting positions.

### Tissue localization and expression pattern

The tissue expression pattern for the CCK receptor genes in Atlantic salmon differed substantially. We found that CCK-1R and CCK-2R1/gastrin had a higher expression in tissues connected with direct regulation of gastrointestinal events than CCK-2R2 and that CCK-2R2 had its highest expression in brain. The spatial expression patterns of different salmon receptors analyzed in this study can substantiate the hypothesis of functional conservation during evolution of these molecules with their ortholog counterparts. Murashita et al. (2009) have reported the mRNA distribution of two CCK peptides in Atlantic salmon namely CCK-N and CCK-L. Phylogenetic analysis suggested that CCK-N and CCK-L belonged to the CCK-1 and CCK-2 gene families respectively. Interestingly, salmon CCK receptors also follows similar evolutionary patterns.

CCK-1R is reported to be involved in satiation via links to peripheral nervous system and vagus nerve. The most accepted vertebrate scheme is that the nutrients enter duodenum and stimulate CCK secretion, some of the CCK acts in a local paracrine manner to stimulate CCK-1R on the sensory fibers of the vagus nerve (Woods 2004). In mammals, CCK-1R is involved in both endocrine and exocrine functions of pancreas, in the regulation of GI motility and gallbladder contractions and it controls the release of enzymes such as somatostatin and pepsinogen from gastric mucosa (Lee et al. 1993; Crawley and Corwin 1994; Wank 1995). Available data suggest that CCK-8 released in the CNS after a meal is responsible for the postprandial increase in colonic motility and that these effects are mediated through activation of central CCK-1R (Gue and Buenno 1991; Gouldson et al. 2000; Lin et al. 2000). A recent study on yellow-tail fish reported that the expression pattern of CCK-1R in gut is similar to that of humans (Furutani et al. 2013). Furthermore, these authors observed that CCK stimulates CCK-1R mediated gall bladder contraction. In this study, the mRNA expression profile of CCK-1R gene in gut tissues of Atlantic salmon are consistent with these findings.

CCK receptors act as gateway of diverse cellular event such as CNS-related functions, growth, proliferation, and inflammatory responses depending on their location and metabolic states (Nagata et al. 1996; Yassin 1999; Piiper et al. 2003; Luyer et al. 2005). In brain, CCK-2R has been demonstrated to participate in cognitive process, release of neurotransmitter and other CNS-related functions (Tullio et al. 2000). Interestingly, CCK-2R2 in brain was found to be much higher expressed than the other two receptors identified in this study. Further studies required to understand the functional distinction (if any) between CCK-2R1 and CCK-2R2.

### Perspectives and significance

In the present study, three CCK-R receptor genes have been identified and cloned from brain and gut tissues of Atlantic salmon. Within gut segments, CCK-1R mRNA were highly expressed in pancreas whereas CCK-2R1/gastrin expression was more abundant in the midgut. The higher levels of expression of CCK-2R2 in brain compared to other organs, together with the observation that it is the most abundant transcript among the three CCK-R genes analyzed in this tissue, is of particular interest and further studies are required to establish its distinct roles in physiological brain functions. Here we also described the major structural and functional motifs of these receptors and showed that they are conserved to that of mammals. Overall, our studies provide a relevant contribution toward the understanding of receptor-mediated execution of CCK-R biological responses in Atlantic salmon, and give an essential foundation to further elucidate by comparative studies the potential conserved roles of these hormone receptors during vertebrate evolution.
